# Two Statistical Tools for Assessing Functionality and Protein Characteristics of Different Fava Bean (*Vicia faba* L.) Ingredients

**DOI:** 10.3390/foods10102489

**Published:** 2021-10-18

**Authors:** Siddharth Sharan, Jens Zotzel, Johannes Stadtmüller, Daniel Bonerz, Julian Aschoff, Anne Saint-Eve, Marie-Noëlle Maillard, Karsten Olsen, Åsmund Rinnan, Vibeke Orlien

**Affiliations:** 1 Department of Food Science, University of Copenhagen, 1958 Frederiksberg C, Denmark; ko@food.ku.dk (K.O.); aar@food.ku.dk (Å.R.); vor@food.ku.dk (V.O.); 2 Paris-Saclay Food and Bioproduct Engineering Research Unit (UMR SayFood), Université Paris-Saclay, INRAE, AgroParisTech, 91300 Massy, France; anne.saint-eve@inrae.fr (A.S.-E.); marie-noelle.maillard@agroparistech.fr (M.-N.M.); 3 Döhler GmbH, 64295 Darmstadt, Germany; Jens.Zotzel@doehler.com (J.Z.); Johannes.Stadtmueller@gmx.de (J.S.); Daniel.Bonerz@doehler.com (D.B.); Julian.Aschoff@doehler.com (J.A.)

**Keywords:** PCA, Pearson’s correlation, processing, foam, emulsion, beverage application

## Abstract

Fava bean (*Vicia faba* L.) is a promising source of proteins that can be potentially used as nutritional and/or functional agents for industrial food applications. Fava ingredients are industrially produced, modified, and utilized for food applications. Their processing conditions influence physico-chemical protein properties that further impact ingredient functionality. To design a functionally suitable ingredient, an understanding of the interrelationships between different properties is essential. Hence, this work aimed to assess two statistical analytical tools, Pearson’s correlation and Principal Component Analysis (PCA), for investigating the role of the process conditions of fava ingredients on their functional and protein properties. Fava concentrates were processed by pH (2, 4, 6.4 and 11), temperature (55, 75 and 95 °C) and treatment duration (30 and 360 min) into different modified ingredients. These were utilized under two application conditions (pH 4 and 7), and their foam and emulsion properties as well as their ingredient characteristics (charge, solubility, and intrinsic fluorescence) were measured. The results show that foam and emulsion properties are not correlated to each other. They are associated with different protein and non-protein attributes as fava concentrate is a multi-component matrix. Importantly, it is found that the results from the two statistical tools are not fully comparable but do complement each other. This highlights that both statistical analytical tools are equally important for a comprehensive understanding of the impact of process conditions on different properties and the interrelationships between them. Therefore, it is recommended to use Pearson’s correlation and principal component analysis in future investigations of new plant-based proteins.

## 1. Introduction

The popular and increasing demand for plant-based foods amongst consumers brings forth the need to understand plant-protein ingredients’ properties, including their functionality for food applications. Fava bean is a promising pulse source of proteins for human consumption, but contains a mixture of non-protein constituents including lipids, starch, dietary fibers and anti-nutritional factors [1,2]. Prior to its use, the whole fava bean must be processed into ingredients such as flours, concentrates, and isolates, which may be further modified through industrial processing. Ingredient fabrication and ingredient modification impact ingredient functional properties, and thus must be optimized using appropriate process conditions and levels along with suitable assessment tools.

The protein-associated functionalities, foaming and emulsification, play a key role in beverage applications. While foams are formed from adsorbed air-in-water (A/W) interfaces, most food emulsions are produced from oil-in-water (O/W) interfaces. Typically, they both need surfactants such as proteins to stabilize the two immiscible phases. However, differences may occur due to changes in the protein functionality and/or effectiveness due to variations in the dispersed phase, its interaction capability, and/or modifications in the protein during application [3,4,5]. In fava beans, various protein types exist in different conformations, and any changes in these conformations during ingredient fabrication, modification and utilization affect the functionality of the ingredient [2,6,7]. In addition, various non-protein constituents, lipids, starch, and dietary fibers are also present in the ingredients [1,2] and may affect how the ingredient functions in a food matrix. Different methodological tools can be used to evaluate ingredients, and various instrumental analyses are used to measure the physico-chemical protein properties and ingredient functionalities, resulting in a myriad of results. These data can be examined individually and provide in-depth information of each individual aspect. Connecting all results may, on the other hand, give a complementary insight into the relationships between properties and functionalities. However, it is difficult to overview many results; thus, statistical data analysis may facilitate the interpretation and assessment of such interrelationships and establish models for choosing raw materials and ingredient processing conditions. This will rely on the reliability of the model; thus, it is essential to be able to correctly evaluate a large dataset. Consequently, a properly estimated correlation model will showcase the complex relationship between protein properties and ingredient functionalities.

This paper aims to compare two different data analytical tools, Pearson’s correlation analysis and Principal Component Analysis (PCA), in their assessment of a large data set. These advanced and relevant statistical tools were chosen for their diverse nature in explaining relationships between variables: one through covariance (PCA) and the other through correlation. Despite being advanced, they can now be easily used through available software and thus are relevant to both industries and researchers working on large data sets [8,9]. By virtue of these tools, the relationship between physico-chemical protein properties (solubility, zeta potential, and intrinsic fluorescence) and ingredient functionalities (foaming and emulsification) of fava bean concentrates is evaluated. The properties measured were modified by different ingredient process conditions (pH, temperature, and treatment duration), and the functionalities were evaluated at two different pHs during utilization.

## 2. Materials and Methods

### 2.1. Ingredient Modification

Fava bean protein concentrate containing 65% proteins (*w*/*w* d.b.) was procured by Döhler GmbH by milling of dried and dehulled beans followed by air classification [10]. This initial concentrate was then modified by the following method: 20% (*w*/*w*) suspensions were prepared with deionized water and stirred for 30 min at 500 rpm using an overhead dissolver stirrer (IKA Works, Inc., Staufen, Germany), followed by pH adjustment (pH_process_) to 2, 4 or 11 using 6 N hydrochloric acid or 3 N sodium hydroxide (Sigma Aldrich, St. Louis, MO, United States) and further stirred for 30 min at 500 rpm. Additionally, a series with the natural suspension pH was used (pH_process_ 6.4), which was also stirred for 30 min at 500 rpm. The suspensions were then heated (T_process_) in a temperature-controlled bath (Lochner Labor+Technik GmBH, Berching, Germany) maintained at 55, 75 or 95 °C and agitated at 700 rpm for a duration (t_process_) of either 30 or 360 min. All the treatments at pH_process_ 4 were performed in triplicates. In total, 36 different suspensions were produced and frozen at −20 °C, followed by freeze-drying and milling to 0.08 mm mesh size to produce ingredient powders. 

### 2.2. Foaming

All ingredients were, in triplicates, suspended to 1% (*w*/*w*) protein concentration at ambient temperature at two different pH of utilization: pH_utilization_ 4 and 7. 150 mL of these suspensions were whipped mechanically at room temperature using a WMF Mechanical Frother (Württembergische Metallwarenfabrik GmbH, Geislingen, Germany) for 2.5 min and the foam was gently transferred to a graduated cylinder (inner diameter = 48.9 mm and height = 400 mm measured using a digital caliper). Foam height and liquid height were recorded manually to calculate the foam and final liquid volumes. Foaming capacity or FC (%) was calculated as the ratio of volume of foam generated after whipping and initial liquid volume. Foam stability or FS (%) corresponded to the foam capacity measured after 30 min [7].

$$\mathrm{FC}\;(\%)=\frac{\mathrm{Foam}\;\mathrm{Volume}\;\mathrm{at}\;0\;\min}{\mathrm{Initial}\;\mathrm{Liquid}\;\mathrm{Volume}\;}\times 100;\;\mathrm{FS}\;(\%)=\frac{\mathrm{Foam}\;\mathrm{Volume}\;\mathrm{at}\;30\;\min}{\mathrm{Initial}\;\mathrm{Liquid}\;\mathrm{Volume}\;}\times 100$$

### 2.3. Emulsification

All ingredients were suspended in triplicates to 1% (*w*/*w*) protein concentration at ambient temperature at pH_utilization_ 4 and 7. These suspensions were added with palm oil medium chain triglycerides (90:10 *w*/*w*) and homogenized for 1 min at 8000 rpm using T-10 Basic ULTRA-TURRAX homogenizer (IKA Works, Inc., Staufen, Germany) fitted with an S-10N-10G dispersing element. These coarse emulsions were passed twice through a homogenizer (Niro-Soavi NS 1001L Panda, Gea Group, Düsseldorf, Germany) at 200 bars. To prevent microbial growth during storage, the emulsions were pasteurized at 80 °C for 10 min after preparation. The pasteurized emulsions were stored at 4 °C for seven days [11]. The emulsion oil droplet size at days 0, 1 and 7 was characterized using laser light scattering by Mastersizer 3000 (Malvern Instruments Ltd., Malvern, UK) with degassed, deionized water used as the dispersant. The particle size distribution from 0.005 to 5000 µm as a function of volume was recorded followed by the estimation of the volumetric mean diameter (D (4;3)), 97th percentile diameter (D_97_) and median diameter (D_50_). The different representations were significantly correlated (Pearson’s correlation coefficient > 0.900, α = 0.05), and therefore it was decided that it is sufficient to use only one popularly reported measure, D(4;3), to evaluate emulsion capacity and stability [10,12].

### 2.4. Nitrogen Solubility

A 1% (*w*/*w*) protein suspension of all ingredients was prepared in citrate phosphate buffer (0.1 M citric acid, 0.2 M dibasic sodium phosphate) at pH_utilization_ 4 and 7 and stirred for 30 min at ambient temperature to produce modified-ingredient-buffer suspensions. The soluble fraction was separated at 8000 g for 20 min and its total nitrogen content was determined by the Dumas method using Rapid MAX-N Exceed (Elementar, Langenselbold, Germany). The solubility of proteins was determined as the ratio (in %) between the total nitrogen estimated from soluble fraction and the suspension.

### 2.5. Absolute Zeta Potential

Absolute value of the zeta potential of the soluble fractions from the modified-ingredient-buffer suspensions was determined by dynamic light scattering in DTS1070 folded capillary cells equilibrated for 120 s at 25 °C using Zetasizer Nano ZS (Malvern Instruments Ltd., Malvern, UK). 

### 2.6. Intrinsic Fluorescence

The modified-ingredient-buffer suspensions (1% and 0.1% *w*/*w* protein suspensions) were characterized by fluorescence excitation-emission scans using FS-920 fluorescence spectrometer (Edinburgh Instruments Ltd., Livingston, United Kingdom) followed by a dimensionality reduction in the fluorescence map by parallel factor analysis (PARAFAC) [13]. PARAFAC is a rapid and efficient curve resolution tool that helps decompose the fluorescence signals into its individual fluorophores. The scores from the PARAFAC models conform to Beer’s Law [14]. This combination of fluorescence and PARAFAC for explaining intrinsic fluorescence of protein and protein interactions is gaining popularity [15,16,17].

**Figure 1:**
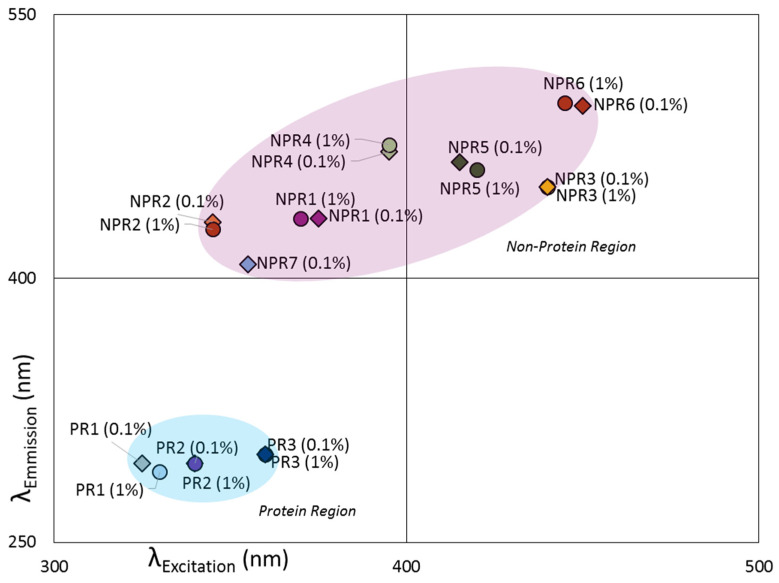
**Fluorescence/PARAFAC Components**: Illustration of the separation of the PARAFAC components based on their maximum excitation and emission wavelengths.

The spectral analysis was performed at both 0.1% (*w*/*w*) and 1% (*w*/*w*) protein concentrations separately. This was due to probable inner filter effects (physical interference) and quenching (chemical interference) observed at 1% concentration [18]. The fluorescence map was obtained by measuring the emission spectra at excitation wavelengths from 250 to 450 nm at 5 nm intervals. The emission spectra were recorded from 300 to 550 nm at 2 nm intervals, with a dwell time of 0.05 s/nm. Slit widths of 5 nm were used for both excitations and emissions, and the iris was set to 100. Rayleigh scattering was removed [19]. The three-way array spectral map obtained was further decomposed by PARAFAC in MATLAB 2017b (Mathworks, Natick, MA, United States) into three matrices: a score matrix, an excitation loading matrix and an emission loading matrix. The landscape was then divided into two areas: one in the protein region, ranging from 250 to 300 nm in excitation and 325 to 360 nm in emission [20,21], and one for the higher region, with excitation between 305 and 450 nm and emission between 362 and 550 nm (Figure 1). A three-component PARAFAC model was sufficient for modelling the protein region (PR1-3). This region is in the range of amino acid residues in proteins (tryptophan, tyrosine and phenylalanine) [20,21,22]. At the same time, it was also necessary for seven components of the secondary region, which hereafter is noted as the non-protein region (NPR1–7). The NPR7 component at 1% (*w*/*w*) protein suspension was removed as it only describes small changes in the spectral behavior of the NPR1 due to inner filter effects. This secondary region explains non-native protein signals from other fluorophores, including vitamins and flavonoids that are inherently present in fava bean [1,22,23,24]. The NPR signals may also contain information on possible protein modifications from Maillard reactions and polyphenol interactions [15,25] (Figure 1).

### 2.7. Correlation Analysis

Pearson’s correlation matrix was generated between all the parameters analyzed for the ingredients in the study, which used Minitab 19.2 (Minitab Inc., State College, PA, USA) for the modified ingredients’ properties.

### 2.8. Principle Component Analysis (PCA)

A PCA model of all the ingredients evaluated at pH_utilization_ 4 and 7 was created using latent variables from the parameters of the functional properties assessed (foaming and emulsification) along with ingredient protein parameters (nitrogen solubility, absolute zeta potential, protein and non-protein PARAFAC components). This was constructed using LatentiX2.12 (Latentix ApS, Frederiksberg, Denmark, www.latentix.com, accessed on: 10 March 2020).

## 3. Results and Discussion

The foam and emulsion properties (capacity and stability) of the 37 ingredients (1 fava bean initial concentrate + 24 modified concentrates + 12 process replicates) at pH_utilization_ 4 and 7 were evaluated against the following attributes: (i) nitrogen solubility, indicating the solubility of proteins; (ii) absolute zeta potential, representing the protein surface charge; (iii) fluorescence PARAFAC components derived from the protein region (PR1–3); and (iv) fluorescence PARAFAC components derived from the non-protein region (NPR1–7). This resulted in a data set of 74 observations by 26 variables.

First of all, it must be taken into consideration that in this study, both PR and NPR signals were considered. It is likely that both these types of signals contribute to the observed functional properties. In complex food systems containing a mixture of components, the modification of non-protein molecules and interactions between these molecules with proteins have been verified earlier [26,27]. PARAFAC can be useful in explaining different chemical components in such systems. For example, cereal flours have been characterized by PARAFAC through a four-component model explaining proteins, vitamins and phenolic acids [24]. Similarly, the presence of phenolic compounds such as caffeic acid, kaempferol and quercetin and fluorophores related to sugar degradation, the Maillard reaction (hydroxymethylfurfural), and carotenoid and chlorophyll degradation have been detected and characterized by PARAFAC in other food systems [14,15,22,24,28]. As this study deals with protein-rich ingredients that have been modified by process conditions, possible interactions between protein and sugars and/or polyphenols could also be expected, leading to changes in protein conformations and availability for functional requirements. Complexes of pulse proteins with phenolic compounds including hydroxycinnamic acids, flavonols, flavones and flavanols have been presented in previous reports [15,29]. The protein–tannin interaction in fava bean has also been reported to modify protein properties. Thus, the NPR signals can be important with respect to functional properties. However, further studies on the chemical nature of these signals would offer stronger insight into the complexity of their interaction. Any relationship found between the signals and their functional properties should encourage a deeper understanding of their involvement in functional properties.

Foaming capacity (FC) and stability (FS) were evaluated against all the sample characteristics using Pearson’s correlation coefficients (Table 1). They were significantly correlated to nitrogen solubility and absolute zeta potential. They, however, correlated differently to different protein and non-protein fluorescence signals measured at different protein concentrations. For instance, FC correlated strongly to PR1, 2 and 3 fluorescence signals at 0.1% protein suspensions, NPR3, 4 and 5 at 0.1% protein suspensions and NPR1, 3 and 5 at 1% protein suspension. On the other hand, FS correlated significantly to PR1, 2 and 3 at 1% protein suspension along with NPR2, 4, and 7 at 0.1% and NPR1, 4, and 6 at 1% protein suspensions. It is interesting to note that the protein region at the low-concentration (0.1%) suspension is more related to the foaming capacity, while the high-concentration (1%) suspension is more related to the foaming stability. The correlation between the NPR fluorescence components to foam capacity and stability suggests the possibility of non-protein components influencing foaming, as a function of the process conditions. 

Emulsification was also tested against different protein and non-protein features. Emulsion oil droplet sizes obtained at three-time intervals (days 0, 1 and 7) were also evaluated against the sample characteristics using Pearson’s analysis. The D(4;3)-value represented the extent of flocculation of oil droplets and possible protein aggregation in the emulsions, thus indicating inversely the capacity of the proteins to form emulsions (day 0) and their capability to stabilize the emulsions (day 7). The emulsion capacity (D(4;3)_Day0_) was significantly correlated with nitrogen solubility and absolute zeta potential, but the D(4;3)_Day7_ after storage was significantly correlated only to the absolute zeta potential (Table 1). A negative correlation between D(4;3) and the two properties indicates that higher protein solubility and absolute zeta potential resulted in decreased emulsion flocculation and protein aggregation. Other significant factors associated with D(4;3)_Day0_ were the PR1, 2, and 3, and NPR2, 4, 5 and 6 for 0.1% suspensions and PR1 and 3, and NPR4 and 5 for 1% suspensions. Just as the case of protein solubility, the D(4;3)_Day7_ after storage of the emulsions was no longer associated with PR1 at 0.1% protein concentration. In general, the set of correlation parameters associated with emulsification is similar, while the correlation parameters differ considerably for the foaming. This indicates that the emulsion capacity and stability are highly correlated, while the two foaming parameters behave differently. 

Overall, different correlations between foam and emulsion properties with nitrogen solubility and absolute zeta potential were obtained by Pearson’s analysis (Table 1). This indicates, that the two beverage functionalities work by different mechanism(s), as supported by the lack of correlation between them (Table 2). Differences in the dispersed phases between food foams (air) and emulsions (oil or water), and the differences in the molecular mechanisms of interaction of proteins with these phases have been suggested in other works [3,30,31]. Studies on ingredients derived from chickpea, lupin, pea, lentil and fava beans have been performed where relationships between protein properties (surface charge, solubility, and intrinsic fluorescence) and foam and emulsion properties have been established to a certain degree [11,32,33,34]. From the previous studies and the results presented, one could infer that it may not be a single property, but a combination of different associated properties that can better explain the underlying mechanism and properties of protein functionalities. For example, from the absolute zeta potential, it could be inferred that despite the protein surface charge representing the amphiphilic behavior of the proteins, further understanding is required to validate how this property helps the protein interact with the distinctive dispersed phases to enable different functional properties. However, it is not within the scope of the present paper to explain the specific physicochemical properties of the functionalities.

**Figure 2:**
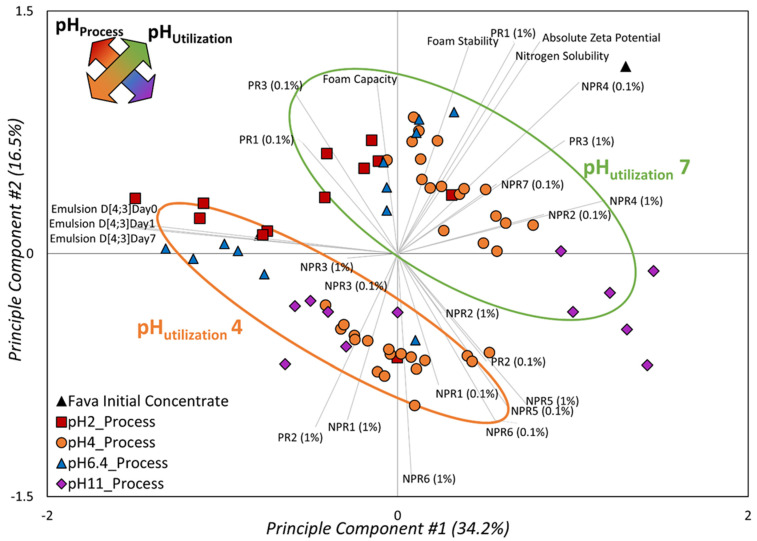
**Principal Component Analysis**: PCA biplot of fava ingredients (1 fava bean initial concentrate + 36 modified concentrates) evaluated at two conditions (pH 4 and pH 7) as scores, with the foam and emulsion functionalities and other ingredient attributes as loadings. The effect of pH during modification is shown by different symbols. The pH during utilization process is indicated with confidence ellipses (α = 0.95). PR and NPR are the PARAFAC components (at 0.1% and 1%, Table 1) based on the protein and non-protein regions of the fluorescence landscape.

The results of the principal component analysis are shown as a biplot of scores and loadings in Figure 2. The PCA scores were separated at two levels: primary separation by different pH_utilization_ 4 and 7, and secondary separation by pH_process_ (2, 4, 6.4 and 11) during ingredient modification. This indicates that both the pHs during ingredient processing and application have an important effect and explain about 51% of variance between different properties. The first two PCA components explained the major variance in the data and, as seen in Figure 2, efficiently described the system with regard to both pH_utilization_ and pH_process_. As seen below, the functionalities did not correlate with each other, whereas the emulsion properties are more correlated than the foaming parameters. Furthermore, the pH_utilization_ mostly influenced foaming, and in particular the FS. The pH_process_, on the other hand, has the largest impact on the differences in emulsion properties. This can be seen as the difference in the pH_utilization_ is from first to third quadrant, with the foam properties mainly moving samples along the second principal component. The emulsion properties are all along the first principal component, the main direction of the difference between the pH_process_ of the samples. 

The PCA showed differences in characteristics associated with foam (FC and FS) and emulsion (D(4;3)_Day0, 1 and 7_) properties. However, comparing this PCA with the Pearson’s correlation (Table 1), it is seen that they do not lead to the exact same conclusion, as the Pearson’s correlation is a pair-wise comparison of variables, while the PCA takes into account all variables at the same time. Furthermore, the correlation pattern seen in the Pearson’s results is not totally explained by the PCA, with the PCA describing around half of the variance (while the Pearson’s correlation is based on all the variance in each of the pair-wise estimates). The PCA is a variance analysis and it clearly indicates that the main variability in the data is due to the two pH parameters (process and utilization). Therefore, it is necessary to investigate both the PCA and the Pearson’s correlation for a successful interpretation. For example, the absolute zeta potential was significantly correlated to FC, FS, D(4;3)_Day0_ and D(4;3)_Day7_ (Table 1), while in the PCA biplot this association is not clear for the emulsion properties (Figure 2). However, through a closer look at the values in Table 1, it becomes clear that the highest correlation is between FC and the zeta potential, while the emulsion properties are negatively correlated (which also is seen in the PCA). On the other hand, the PCA shows a clear association between absolute zeta potential and the pH_utilization_. Therefore, an overall interpretation from the two analyses suggests that foam and emulsion properties are strongly correlated to the zeta potential and nitrogen solubility (Table 1), and they are all influenced by process conditions, especially pH during ingredient utilization (Figure 2). Relationships between charge and solubility, and foam and emulsion properties have been indicated in plant-based ingredients. In fact, the same negative correlation between higher surface charge and decreased emulsion droplet size has been noted for chickpea, fava, pea and lentil isolates [11]. A lower absolute charge is often related to a lower solubility, and the protein intrinsic fluorescence is often used to characterize protein hydrophobicity and the folded nature. Process conditions changing protein properties have been shown to modify foam and emulsion properties [35]. Figure 2 and Table 1 clearly illustrate these different relationships between process conditions, changes in protein and non-protein aspects, and thereby changes in foam and emulsion properties.

The two analyses associating functionalities to the fluorescence signals indicate that fava bean concentrate is a multi-component system containing different proteins and non-protein elements as well as protein modifications, which all seem to affect functional properties. These fluorescence signals (PR and NPR) were associated with functionalities (Table 1) and were highly impacted by the pH during ingredient modification and utilization (Figure 2). The two separate PARAFAC models of the fluorescence data (at 0.1% and 1% *w*/*w*) both gave three underlying components in the protein region, but different components (seven and six components at 0.1% and 1%, respectively) in the non-protein region. Additionally, these signals were highly affected by the dilution. For instance, the PR components at 0.1% and 1% protein suspensions were differently correlated to functionalities. This clearly indicates the possibility of inner filter effects in the fluorescence data, most probably more pronounced at the 1% suspension than at the 0.1% one. Despite this probable inner filter effect in the 1% suspension, it is of interest to note that the data from 1% suspension are more related to the foaming, and thus also to pH_utilization_, while the data from 0.1% are more related to the emulsion properties, and thus also to pH_process_.

## 4. Conclusions

Statistical models facilitated a rapid comprehension of the large data set that represented functional and physico-chemical properties. Beverage functionality, as measured by the foam and emulsion properties of different fava bean ingredients modified by various process conditions, was correlated to their multi-component character. These two beverage functionalities were first and foremost not correlated to each other. The associations between the ingredient characteristics and functionalities obtained by Pearson’s correlation analysis and PCA were not fully comparable as one explained association and the other suggested causalities and effects. Where Pearson’s correlation validated the associations between functionalities and physico-chemical properties, PCA suggested the impact of process conditions on ingredient properties along with some obvious associations between the properties. Despite certain breakthroughs in the critical understanding of research methods, we must note that further investigations are needed to identify and explain the underlying phenomena in the ingredient responsible for the functionality. In this respect, a paper focusing on the mechanistic understanding of the results presented in this paper is under preparation.

## Figures and Tables

**Table 1:** Pearson’s correlation analysis between foam (FC and FS) and emulsion (D(4;3)) properties and protein and non-protein features.

	Foaming		Emulsification	
**Ingredient Properties**	FC	FS	D(4;3)_Day0_	D(4;3)_Day7_
Nitrogen Solubility	0.284 *	0.495 *	−0.291 *	−0.271
Absolute Zeta Potential	0.343 *	0.693 *	−0.357 *	−0.366 *
PR1 (0.1%)	0.288 *	−0.004	0.240 *	0.227
PR2 (0.1%)	−0.305 *	0.035	−0.199 *	−0.187 *
PR3 (0.1%)	0.367 *	0.166	0.339 *	0.312 *
PR1 (1%)	0.203	0.447 *	−0.277 *	−0.286 *
PR2 (1%)	−0.137	−0.271 *	0.221	0.213
PR3 (1%)	0.223	0.404 *	−0.436 *	−0.455 *
NPR1 (0.1%)	−0.078	−0.040	−0.041	−0.030
NPR2 (0.1%)	0.197	0.493 *	−0.370 *	−0.321 *
NPR3 (0.1%)	0.274 *	0.149	0.058	0.066
NPR4 (0.1%)	0.324 *	0.678 *	−0.515 *	−0.477 *
NPR5 (0.1%)	−0.228 *	−0.096	−0.428 *	−0.378 *
NPR6 (0.1%)	0.093	−0.015	−0.254 *	−0.233 *
NPR7 (0.1%)	0.207	0.329 *	−0.184	−0.149
NPR1 (1%)	−0.261 *	−0.543 *	0.162	0.121
NPR2 (1%)	−0.015	−0.123	−0.066	−0.073
NPR3 (1%)	0.339 *	0.119	0.184	0.187
NPR4 (1%)	0.196	0.526 *	−0.578 *	−0.543 *
NPR5 (1%)	−0.276 *	−0.016	−0.453 *	−0.394 *
NPR6 (1%)	−0.170	−0.309 *	−0.009	−0.015

Significant differences are indicated by bold and * (α = 0.05).

**Table 2:** Pearson’s correlation analysis between foaming and emulsification.

	Foam Capacity	Foam Stability
Emulsion Capacity, D(4;3)_Day0_	0.165	−0.099
Emulsion Stability, D(4;3)_Day7_	0.172	−0.054

## Data Availability

The data has been shared in Zenodo data repository supported by the European Commission (Framework Programme 7/Horizon 2020).
